# High-flow arteriovenous fistula in X-linked Alport syndrome: a case report

**DOI:** 10.3389/fmed.2023.1227283

**Published:** 2023-10-19

**Authors:** Daisuke Takahashi, Kan Katayama, Yoshinobu Iyoda, Ayumi Fukumori, Kayo Tsujimoto, Masahiro Yamawaki, Fumika Tanaka, Ryosuke Saiki, Keiko Oda, Yasuo Suzuki, Tomohiro Murata, Yoshinaga Okugawa, Kaoru Dohi

**Affiliations:** ^1^Department of Cardiology and Nephrology, Mie University Graduate School of Medicine, Tsu, Japan; ^2^Tsu Minami Clinic, Tsu, Japan; ^3^Department of Genome Medicine, Mie University Hospital, Tsu, Japan

**Keywords:** Alport syndrome, *COL4A5* mutation, arteriovenous fistula, heart failure, high-flow access, high-output cardiac failure, radial artery banding

## Abstract

Most male X-linked Alport syndrome patients with *COL4A5* nonsense mutations experience end-stage kidney failure by 30 years old. Although there is no definition of high-flow arteriovenous fistula, access blood flows greater than 2000 mL/min might predict the occurrence of high-output heart failure. A 50-year-old Japanese man had suffered from proteinuria at 4 years old and sensorineural hearing loss and a lenticular lens at 20 years old. He had started to receive hemodialysis treatment due to end-stage kidney disease at 22 years old. A genetic test confirmed a novel hemizygous nonsense variant *COL4A5* c.2980G > T (p.Gly994Ter), and he was diagnosed with X-linked Alport syndrome. *COL4A5* c.2980G > T was considered “pathogenic” according to the American College of Medical Genetics and Genomics guidelines and *in vitro* experiments. Shortness of breath on exertion was exaggerated, his brachial artery blood flow was over 4,236–4,353 mL/min, his cardiac output was 5,874 mL/min, and he needed radial artery banding at 51 years old. After radial artery banding surgery, the brachial artery blood flow decreased to 987–1,236 mL/min, and echocardiography showed a cardiac output at 5100 mL/min with improved E’ and E/E’. His shortness of breath on exertion improved gradually. Although rare, high-output heart failure due to high-flow arteriovenous fistula should be kept in mind as a complication in X-linked Alport syndrome patients, and our patient was successfully treated with radial artery banding surgery.

## Background

Alport syndrome (AS) is characterized by ocular abnormalities, sensorineural deafness, and progressive kidney failure (1, 2). The major hereditary form of AS is X-linked AS caused by a *COL4A5* mutation, and 90% of affected male patients with a *COL4A5* nonsense mutation develop end-stage kidney failure by 30 years old (3). However, the outcomes of kidney replacement therapy for AS are reported to be favorable (4, 5).

Although there was no definition of high-flow arteriovenous fistula (AVF), access blood flows (Qa) greater than 2000 mL/min could predict the occurrence of high-output heart failure (HOHF) (6). Twenty-six percent of the kidney-transplanted patients had to receive AVF closure due to symptoms of HOHF, and their preoperative mean Qa value was 2,197 mL/min (7). The prevalence of high-flow AVF in another article, defined as a Qa >2,000 mL/min, was 24% (8).

We herein report a case of X-linked AS (XLAS) with high-flow AVF that was successfully treated with radial artery banding.

## Case presentation

A 50-year-old Japanese man had had proteinuria at 4 years old and been diagnosed with AS by a kidney biopsy at 8 years old. He developed sensorineural hearing loss and astigmatism due to a lenticular lens at 20 years old. He started to receive hemodialysis treatment due to end-stage kidney disease (ESKD) at 22 years old. At his presentation, he was currently being treated with 20 μg of darbepoetin alpha once weekly and intravenous iron as needed for renal anemia. For secondary hyperparathyroidism, 2.5 mg of maxacalcitol was being administered intravenously 3 times a week, and 4 mg of oral evocalcet was also being used. A hearing aid had been installed for sensorineural hearing loss at 48 years old.

**Figure 1:**
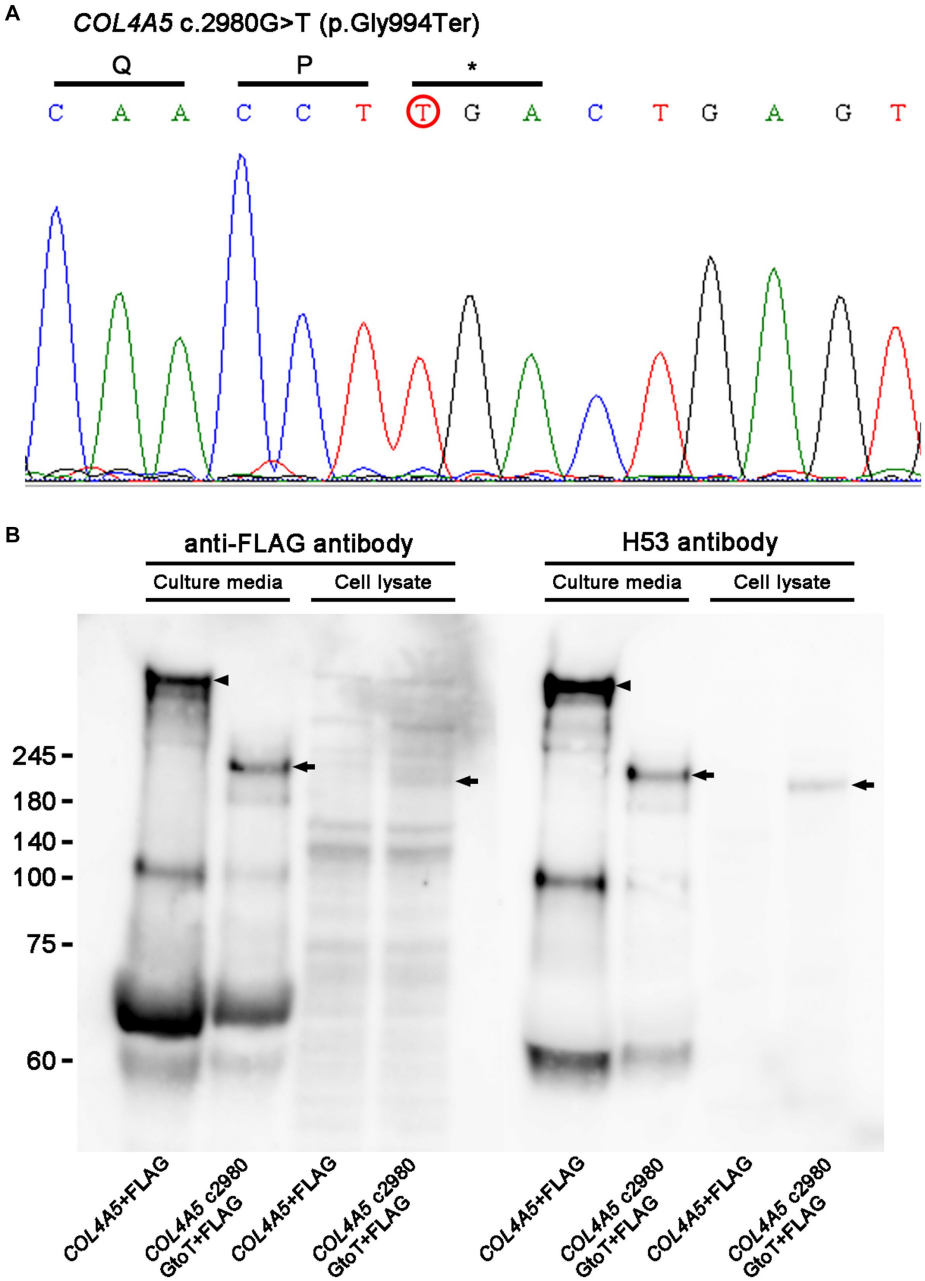
**(A)** Sanger sequencing. A genetic analysis identified a hemizygous nonsense variant of *COL4A5* c.2980G > T (p.Gly994Ter; red circle). **(B)** Western blot analysis results. While full-length COL4A5 + FLAG protein (arrowheads) was detected only in culture media, not in cell lysate transfected with pcDNA3-*COL4A5* + FLAG, shorted bands (arrows) were detected in culture media and cell lysates transfected with pcDNA3-*COL4A5* c2980GtoT + FLAG. Similar results were obtained with rabbit polyclonal anti-FLAG antibody and rat monoclonal H53 antibody, which targeted the COL4A5 protein.

A genetic test confirmed a hemizygous nonsense variant *COL4A5* c.2980G > T (p.Gly994Ter; Figure 1A), and he was diagnosed with XLAS. His mother was also undergoing hemodialysis at another hospital, but no genetic testing was performed due to a lack of consent. *COL4A5* c.2980G > T was considered “pathogenic” according to the American College of Medical Genetics and Genomics (ACMG) guidelines (9) and seemed to be a novel variant, since there was no report in the ClinVar database or Leiden Open Variation Database (LOVD) (10).

To examine the protein function of the variant, a mutant *COL4A5* construct was made with a QuikChange Lightning Site-Directed Mutagenesis Kit (Agilent Technologies, Santa Clara, CA, United States). The mutagenesis primers were as follows: forward GCCTGGAGACCCAGGGCAACCTtGACTGAGTGGACAACCTGG, reverse CCAGGTTGTCCACTCAGTCaAGGTTGCCCTGGGTCTCCAGGC. pcDNA3-*COL4A5* c2980GtoT + FLAG was generated from pcDNA3-*COL4A5* + FLAG as a template (11). Either pcDNA3-*COL4A5* + FLAG 7 μg or pcDNA3-*COL4A5* c2980GtoT + FLAG 7 μg was transfected with Lipofectamine 3,000 (Thermo Fisher Scientific, Waltham, MA, United States) in human embryonic kidney 293 (HEK293) cells cultured with Dulbecco’s modified Eagle’s medium (DMEM; Fujifilm Wako, Tokyo, Japan) with 10% bovine serum albumin (BSA) in 6-cm dishes for harvesting cells or culture media. Transfected cells were harvested the next day, and cell lysates were made by adding LDS sample buffer (Thermo Fisher Scientific) with 10% dithiothreitol.

For harvesting culture media, transfected cells were cultured in DMEM without BSA for 4 days, and the culture media were concentrated with an Amicon Ultra15 Centrifugal Filter (Merck Millipore, Burlington, MA, United States) at 3,000 rpm for 20 min. Samples for a Western blot analysis from the concentrated media were made by adding LDS sample buffer with 10% dithiothreitol. The same amount of culture media or cell lysates were loaded in 4–12% gels in MES buffer, and gel electrophoresis was performed at 200 volts for 70 min, followed by transfer at 20 volts overnight. The next day, blocking was performed in Tris-buffered saline with 0.1% Tween 20 and 5% milk at room temperature for 30 min, followed by incubation with primary antibodies, such as rabbit polyclonal anti-FLAG antibody (11,000 dilution; Sigma Aldrich, St. Louis, MO, United States) or rat monoclonal H53 antibody (1,100 dilution), which targeted the COL4A5 protein, overnight (12). The next day, the membrane was developed with ECL prime (Figure 1B). Experiments with mutant *COL4A5* construction revealed that mutant *COL4A5* was less strongly secreted extracellularly than wild-type *COL4A5*.

High-flow AVF with a shunt aneurysm was obvious at 48 years old, and shortness of breath on exertion was exaggerated. Brachial artery blood flow was over 2,000 mL/min, and cardiac output was 6,424 mL/min at 49 years old (Table 1), so shunt reconstruction (reanastomosis in front of the shunt aneurysm) was performed for the shunt aneurysm because he wanted to have the shunt aneurysm removed cosmetically. However, while shunt aneurysm resection was performed 2 and 3 months later, his high-flow AVF persisted. His brachial artery blood flow was over 4,236–4,353 mL/min, and cardiac output was 5,874 mL/min with decreased E’ and elevated E/E’ and needed an operation at 51 years old (Table 1).

**Table 1:** Chronological changes in echographic parameters.

Parameters	2020	2021	2022	2022 After operation
Ejection fraction (%)	60	57	50	49
Stroke volume (ml)	88	81	89	75
Heart rate (/min)	73	78	66	68
E (m/s)	0.7	0.7	1	0.6
A (m/s)	0.8	0.7	0.9	0.7
E’ (cm/s)	4.8	6.3	6	8.5
E/A	0.86	0.97	1.09	0.96
E/E’	14.7	10.3	15.7	7.6
Cardiac output (ml/min)	6,424	6,318	5,874	5,100
Brachial artery blood flow (ml/min)	>2000	2,619–3,374	4,236–4,353	987–1,236
Resistive index	0.56	0.61–0.64	0.47–0.52	0.47–0.6
Anastomosis vein diameter (mm)	NA	9	12.2	6.7

NA, not available.

**Figure 2:**
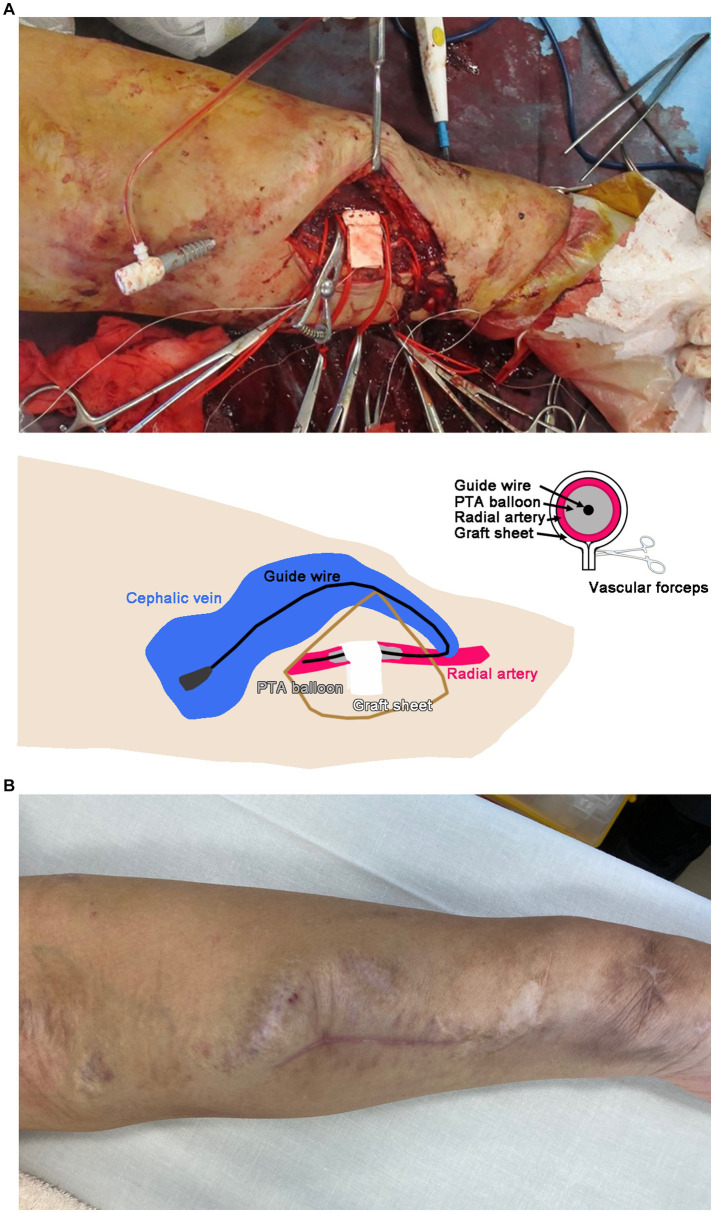
**(A)** Banding operation of the left radial artery with a graft sheet. A schematic illustration is shown in the lower panel. The suture site was determined using vascular forceps. PTA, percutaneous transluminal angioplasty. **(B)** Left forearm 3 months after the operation.

The operation procedure was as follows: The radial artery near the AVF was exposed by a 5-cm skin incision. The radial artery was dilated to 7 mm in diameter, and its wall was calcified and thinned. The distal side of the radial artery was found to be closed in the previous shunt reconstruction for the shunt aneurysm. Next, a rectangular sheet of 5-mm-diameter polytetrafluoroethylene (PTFE) graft opened to a length of 5 cm was wrapped around the radial artery under the guidance of a 5-mm-diameter percutaneous transluminal angioplasty (PTA) balloon catheter through the cephalic vein (Figure 2A). Once the vascular forceps had been snapped (lower panel, Figure 2A), the PTA balloon was deflated and the blood flow in the brachial artery was examined. Since the blood flow in the brachial artery decreased from 4,000 to 1,000 mL/min after suturing, the wound was closed. When the brachial artery blood flow was measured again, it was 1,500–2000 mL/min, and the suturing was judged to be insufficient. The strength of the suturing was adjusted by covering the second rectangular sheet of a 5-mm-diameter PTFE graft on the first sheet. The wound was then closed to adjust the brachial artery blood flow to 700–800 mL/min. The final blood flow was 800 mL/min.

The patient’s shortness of breath on exertion gradually improved, and the dilation of the cephalic vein in his left forearm also improved by 3 months after the operation (Figure 2B). Brachial artery blood flow was decreased to 987–1,236 mL/min, and echocardiography showed cardiac output at 5100 mL/min with improved E’ and E/E’ values (Table 1).

## Discussion and conclusions

We experienced a case of XLAS with a novel hemizygous nonsense variant in *COL4A5,* and cell experiments demonstrated that the mutant *COL4A5* construct was partially retained in the cell lysate and secreted insufficiently in the culture media compared to the wild-type *COL4A5* construct. The lack of production of the wild-type COL4A5 protein was the primary origin of XLAS in the present case. In addition, retention of the mutant COL4A5 protein in podocytes may cause endoplasmic reticulum stress activation in podocytes (13). Therefore, *COL4A5* c.2980G > T was considered “pathogenic.” The patient had received an AVF operation due to ESKD at 22 years old. As he was not willing to undergo peritoneal dialysis and had no chance of receiving living or deceased kidney transplantation, he continued to receive hemodialysis treatment. He developed HOHF due to high-flow AVF 26 years old after AVF construction, and his symptoms improved with radial artery banding.

While major complications of AVF include aneurysms, infections, steal events, thrombotic events, and venous hypertensive events (14), HOHF due to high-flow AVF should be kept in mind as a rare complication, as most XLAS male patients develop ESKD by 30 years old, and high-flow AVF can occur due to a lack of arteriosclerosis. A previous report revealed hereditary nephropathy in 19 of 113 kidney transplant patients (16.8%), and 5 of these 19 patients (26.3%) required shunt intervention (7). The percentage of shunt intervention was 25.7% (29 out of 113 patients), regardless of underlying kidney disease (7). Although reconstruction of the AVF on the contralateral side was considered in the present case, the patient did not wish to receive it. There are various methods other than radial artery banding for suppression of high-flow AVFs (Table 2) (15–19). A previous report showed 44 (29.7%) postoperative complications (13 recurrences of excessive blood flow, 3 decreased blood flow, 23 shunt occlusions, 2 PTA, 3 graft infections) in 148 patients with high-flow AVF (20). The reasons why this procedure was selected in the present case were as follows: first, the diameter of the AVF was large; second, frequent surgeries might have caused strong adhesions, making it difficult to adjust the blood flow on the AVF side; and third, with radial artery banding, we could monitor the brachial artery blood flow during the operation. The present surgery was considered a modified version of AVF inflow artery banding. For blood flow control surgery for high-flow AVF, a treatment method suitable for individual situations should be selected. Banding surgery in the present case improved the brachial artery blood flow, cardiac output, E’, and E/E’ under echography as well as the patient’s shortness of breath during exercise.

**Table 2:** Blood flow suppression techniques.

**AVF banding**
AVF outflow vein banding
Suturing at the AVF anastomosis/draining vein
AVF inflow artery banding [artificial blood vessel banding, MILLER method (15)]
AVF inflow artery banding + inflow artery peripheral ligation
Anastomotic septoplasty
**AVG banding**
Graft interposition
Distal revascularization and interval ligation (DRIL) (16)
Revision using distal inflow (RUDI) (17)
Distal ligation of the inflow artery anastomosis
Central ligation of inflow artery
**Graft streamer method**
Graft inclusion technique (18)
Graft covering technique

AVF, arteriovenous fistula; AVG, arteriovenous graft; MILLER, Minimally Invasive Limited Ligation Endoluminal-assisted Revision.

A previous study compared gene expression analyses of the venous segment of high-flow AVF with normal AVF, and biological developmental processes and glycosaminoglycan binding were mostly upregulated (21). In another study, 74 genes, including mainly those associated with inflammation, were downregulated in cases with high-flow AVF (22); however, an association between *COL4A5* and high-flow AVF was unlikely.

Several limitations associated with the present study warrant mention. Since this is an isolated case report, the findings may not be universally applicable to other patients with XLAS. Longer follow-up of the present patient and larger observational or interventional studies are needed to assess the efficacy of banding surgery. Earlier monitoring prior to the onset of symptoms may have helped prevent HOHF due to high-flow AVF.

In conclusion, we experienced a case of XLAS with high-flow AVF that was successfully treated with banding surgery.

## Data availability statement

The original contributions presented in this study are included in the article/supplementary material, further inquiries can be directed to the corresponding author.

## Ethics statement

The studies involving humans were approved by Institutional Review Board of Mie University Graduate School of Medicine. The studies were conducted in accordance with the local legislation and institutional requirements. The participants provided their written informed consent to participate in this study. Written informed consent was obtained from the individual(s) for the publication of any potentially identifiable images or data included in this article.

## Author contributions

DT, KK, YI, AF, KT, MY, FT, RS, KO, and YS participated in the acquisition of clinical data. DT, KK, YI, TM, YO, and KD carried out the analysis of the patient’s clinical course and data interpretation. DT and KK wrote a draft of the manuscript. YI, AF, KT, MY, FT, RS, KO, YS, TM, YO, and KD revised it critically. All authors contributed to the article and approved the submitted version.

## Abbreviations

ACMG: American College of Medical Genetics and Genomics
AS: Alport syndrome
AVF: arteriovenous fistula
BSA: bovine serum albumin
DMEM: Dulbecco’s modified Eagle’s medium
ESKD: end-stage kidney disease
HEK293: human embryonic kidney 293
HOHF: high-output heart failure
LOVD: Leiden Open Variation Database
PTA: percutaneous transluminal angioplasty
PTFE: polytetrafluoroethylene
XLAS: X-linked Alport syndrome
